# Sialyltransferase ST3GAL1 promotes cell migration, invasion, and TGF-β1-induced EMT and confers paclitaxel resistance in ovarian cancer

**DOI:** 10.1038/s41419-018-1101-0

**Published:** 2018-10-30

**Authors:** Xin Wu, Junda Zhao, Yuanyuan Ruan, Li Sun, Congjian Xu, Hua Jiang

**Affiliations:** 1 Key Laboratory of Female Reproductive Endocrine Related Diseases; The Obstetrics and Gynecology Hospital, Fudan University, Shanghai, 200011 China; 2 First Affiliated Hospital of Xinjiang Medical University, Wulumuqi, 830054 China; 3 Key Laboratory of Glycoconjugate Research Ministry of Public Health, School of Basic Medical Sciences; Department of Biochemistry and Molecular Biology, School of Basic Medical Sciences, Fudan University, Shanghai, 200433 China; 4 The Obstetrics and Gynecology Hospital, Fudan University, Shanghai, 200011 China

## Abstract

Sialyltransferases transfer sialic acid to nascent oligosaccharides and are upregulated in cancer. The inhibition of sialyltransferases is emerging as a potential strategy to prevent metastasis in several cancers, including ovarian cancer. ST3GAL1 is a sialyltransferase that catalyzes the transfer of sialic acid from cytidine monophosphate-sialic acid to galactose-containing substrates and is associated with cancer progression and chemoresistance. However, the function of ST3GAL1 in ovarian cancer is uncertain. Herein, we use qRT-PCR, western blotting, and immunohistochemistry to assess the expression of ST3GAL1 in ovarian cancer tissue and cell lines and investigate whether it influences resistance to paclitaxel in vitro and in a mouse xenograft model. We found that ST3GAL1 is upregulated in ovarian cancer tissues and in the ovarian cancer cell lines SKOV-3 and OVCAR3 but downregulated in A2780 ovarian cancer cells. Overexpression of ST3GAL1 in A2780 cells increases cell growth, migration, and invasion whereas ST3GAL1 knockdown in SKOV-3 cells decreases cell growth, migration, and invasion. Furthermore, overexpression of ST3GAL1 increases resistance to paclitaxel while downregulation of ST3GAL1 decreases resistance to paclitaxel in vitro, and overexpression of ST3GAL1 increases tumorigenicity and resistance to paclitaxel in vivo. Transforming growth factor-β1 can increase ST3GAL1 expression and induce ovarian cell epithelial–mesenchymal transition (EMT). However, knockdown of ST3GAL1 inhibits EMT expression. Taken together, our findings have identified a regulatory mechanism involving ST3GAL1 in ovarian cancer. ST3GAL1 may be a promising target for overcoming paclitaxel resistance in ovarian carcinoma.

## Introduction

Epithelial ovarian cancer is the sixth most frequently diagnosed cancer in women and accounts for ~4% of all cancer-related female mortality^1,2^. Ovarian cancer occurs as four main subtypes: serous, mucinous, endometrioid, and clear cell^3,4^. Of these, the most frequent subtype is serous ovarian cancer, which has a high chromosomal instability owing to the presence of TP53 mutations^4,5^. The TP53 protein is thought to act as a tumor suppressor by regulating cell cycle arrest, apoptosis, and DNA damage repair and can be converted from a tumor suppressor to an oncogene by gain-of-function mutations^6^. Ovarian cancer is difficult to detect due to the absence of specific symptoms in the early stages, therefore, 75% of women are diagnosed at an advanced stage after metastasis has occurred and survival rates are substantially reduced^7^.

Sialyltransferases transfer sialic acid to nascent oligosaccharides and are upregulated in cancer^8^. Moreover, hypersialylation is a consequence of the general upregulation of sialylated glycans on cell surfaces and is a characteristic of tumors. Cancer-associated hypersialylation is thought to influence the interactions of tumor cells and has been associated with metastatic cell behavior including invasion and enhanced cell survival^9,10^. Metastasis is a leading cause of mortality associated with ovarian cancer and mostly involves the genetically unstable high-grade serous carcinoma^5,11^. Therefore, the inhibition of sialyltransferases is a potential strategy in preventing metastasis in several cancers, including pancreatic and ovarian cancer^12^.

Mammalian sialyltransferases are a family of 20 conserved enzymes that are further divided into four subfamilies: ST3Gal, ST6Gal. ST6GalNAc, and ST8SIA^13^. In studies which have focused on epithelial carcinomas, 10 of these 20 sialyltransferases have been associated with the progression of cancer^9^. ST3GAL1 adds a sialic acid in an α2,3 linkage to Gal β1,3 GalNAc. Overexpression of ST3GAL1 leads to an increase in the sialylation of O-glycan Tn to Sialyl-Tn in breast cancer and is associated with the expression of the mucin protein MUC1^14^. MUC1 has been found to be upregulated in ovary carcinomas and is also associated with increased tumor invasiveness^15^. In the initial process of tumorigenesis, an epithelial–mesenchymal transition (EMT) can occur in ovarian carcinoma cells, which is accompanied by a change in the expression of cadherin and integrin^16^. Cancer cells are carried via peritoneal fluid to the abdominal peritoneum or omentum, where they attach and eventually grow into tumor nodules on mesothelium covered surfaces, leading to the possibility of ascites, bowel obstruction, and tumor cachexia^11^.

Resistance to chemotherapy is a contributing factor to mortality in ovarian cancer^17,18^. The mechanisms of chemoresistance in ovarian cancer are unclear but are thought to involve both intrinsic and acquired molecular responses^19^. Intrinsic resistance involves the presences of cancer stem cells whereas acquired resistance involves the genetic and epigenetic alteration of genes in response to repetitive chemotherapy^19,20^. The drugs prescribed most frequently to treat ovarian cancer are platinum-based agents and taxanes^21^. Platinum-based agents, such as cisplatin, induce the formation of crosslinked-DNA adducts, which eventually lead to cell death^22^. Resistance to cisplatin includes changes in multiple cell defense mechanisms by epigenetic and genetic changes which result in the loss of cell surface-binding sites and transporters^23^. Taxanes, such as paclitaxel, function against cancer cells with a different mechanism to platinum-based agents by interfering with microtubules to inhibit cell division^24^. Resistance to paclitaxel (tradename Taxol) is mainly thought to involve upregulated exportation of the drug by increased P-glycoprotein activity to decrease cellular accumulation^25^. Other mechanisms could involve an alteration in the expression of microtubule regulatory proteins or enhanced cell survival associated with hypersialylation^10,26^.

In this work, we examine the role of the sialyltransferase ST3GAL1 in ovarian cancer tissue and the human ovarian cancer cell lines SKOV-3, OVCAR3, and A2780. SKOV-3 is a human ovarian cancer cell line with an epithelial-like morphology which exhibits resistance to tumor necrosis factor and several cytotoxic drugs^27^. OVCAR3 is a cell line established from the malignant ascites of a patient treated for progressive adenocarcinoma of the ovary and is also resistant to cytotoxic drugs^28^. A2780 cells originate from an untreated patient with ovarian cancer^27^. Transforming growth factor-β1 (TGF-β1) is a multifunctional cytokine that has been found to stimulate EMT in epithelial cells^29,30^. We also investigate the expression of ST3GAL1 in EMT induced by TGF-β1 and paclitaxel resistance in ovarian cancer cell lines and a xenograft mouse model.

## Materials and methods

### Cell culture

A human normal ovarian surface epithelial cell line (NOEC) and ovarian cancer cell lines SKOV-3, OVCAR3, and A2780 cells were purchased from the American Type Culture Collection (Manassas, VA, USA).We confirm that the authentication of all cell lines used the full policy and requirements are available in the instructions to authors. SKOV-3, OVCAR3, and A2780 cells were maintained in RPMI1640 supplemented with 10% fetal bovine serum (FBS) and 100 U/mL of penicillin and streptomycin under standard culture conditions. The cultures were maintained at 37 °C in a humidified atmosphere of 5% CO_2_.

### Patients and tissue samples

Ovarian cancer tissues were obtained from 78 patients who had a primary surgical resection in the Obstetrics and Gynecology hospital of the Fudan University,China. The histopathological and TNM stages of tumors can be found in Table 1. Relative normal tissues from 15 patients were used as a control. The Ethics Committee of the Obstetrics and Gynecology hospital of the Fudan University (Shanghai, China) approved the study and prior written consent was obtained from all patients. Tissue samples were frozen in liquid nitrogen after surgery and stored at −80 °C until analysis.

**Table 1:** ST3GAL1 expression and clinicopathological characteristics of 78 patients with ovarian cancer

ST3GAL1				
Parameters	Low (%)	High (%)	Total	*P*-value
Age (years old)				0.507
<50	17 (21.8)	23 (29.5)	40	
≥50	19 (24.4)	19(24.4)	38	
Histopathology			0.501	
Serous	12 (15.4)	21 (26.9)	33	
Mucinous	8 (10.3)	8 (10.3)	16	
Clear cell	8 (10.3)	6 (7.7)	14	
Endometrioid	8 (10.3)	7 (9.7)	15	
TNM stage			0.014^*^	
I	14 (17.9)	7 (9.0)	21	
II	12 (15.4)	10 (12.8)	22	
III	10 (12.8)	25 (32.1)	35	

^*^p < 0.05, *χ*^2^ test

### RNA extraction and quantitative real-time PCR

RNA was extracted from tissues using an RNA isolation kit (Tiangen Biotech, Beijing, China) according to the manufacturer’s instructions. A PrimeScript RT reagent kit (TaKaRa, Shiga, Japan) was used to reverse transcribe 2 μg of total RNA into cDNA and used for quantitative real-time PCR (qRT-PCR). The qRT-PCR was performed in a reaction solution containing 20 ng cDNA, 0.2 μmol/L primers, and 10 μL SYBR Premix Ex Taq (TaKaRa, Shiga, Japan). The primers used were as follows: ST3GAL forward: 5′- TTCCGGGAGCTGGGAGATAA-3′,reverse:5′- CTCACCACCCACTCCAAGTC −3′; Data were normalized to β-actin. The relative quantification of expression was calculated using the 2^−∆∆CT^ method^31^.

### Western blot analysis

Cells were lysed to extract total protein using RIPA lysis buffer and then proteins were separated by SDS-PAGE. After SDS-PAGE, proteins were transferred to PVDF membranes. Membranes were then incubated with primary antibody following manufacturer’s instructions followed by incubation with a secondary antibody. Primary antibodies included a Rabbit Polyclonal antibody against human ST3GAL1 (LS-C185763, LifeSpan BioSciences) at a dilution of 1:1000. GAPDH was used as a control.

### Immunohistochemistry

Xenografted tumor tissues were isolated and fixed in 10% formalin for 24 h, dehydrated and embedded in paraffin and then sectioned with a microtome (Leica, Deerfield, IL). Immunohistochemistry (IHC) was performed on dewaxed and hydrated 4-mm-thick sections of tissue using anti-ST3GAL1 Polyclonal antibody (LS-C185763), diluted 1:300. After blocking with 1% BSA, the sections were incubated overnight at 4 °C with primary antibody followed by incubation with HRP-conjugated secondary antibody for 2 h at room temperature. The percentage of positive cells was scored as: 0, 5%; 1, 25%; 2, 50%; 3, 75%; and 4, 100%.

### Cell proliferation assay

Cell proliferation was quantified using the cell counting kit-8 (CCK-8, Dojindo, Kumamoto, Japan) according to the manufacturer’s instructions. Briefly, 2000 cells/well were seeded into 96-well plates and cultured overnight. We added 10 µl CCK-8 reagent to detect cell proliferation at 1–5 days by a measuring the A450 nm with an Epoch Microplate spectrophotometer (BioTek, Winooski, VT, USA).

### Migration and invasion

SKOV-3 and A2780 cells were plated in the uncoated wells of 24-well inserts for migration assays or Matrigel-coated wells for invasion assays (BD Bioscience, Bedford, MA, USA). In total, 2 × 10^4^ cells/well in 100 μL serum-free medium were added to the upper chamber and 600 μL 10% FBS serum medium was added to the lower chamber as a chemoattractant. After 16 h incubation, non-migrating cells were removed by wiping the upper chamber with a cotton swab on and migrated cells in the lower chamber were quantified in five random fields using a microscope at ×200 magnification (Nikon).

### Animals and xenografts

A xenograft tumor model was generated as previously described. Nude female BALB/cA-nu mice (6-weeks-old) were obtained from Shanghai SLAC Laboratory Animal Co., Ltd. (Shanghai, China; *n* = 6 per group) and were weighed, coded, and randomly assigned to experimental groups. All experiments were approved and performed according to the guidelines of the Ethics Committee of the Obstetrics and Gynecology hospital of the Fudan University (Shanghai, China), conformed to the Principles of Laboratory Animal Care (National Society for Medical Research), and were conducted according to the National Institute of Health guidelines. Tumor formation was assessed in nude mice. A2780 cells (2 × 10^6^) were infected with negative control (NC) or overexpression ST3GAL1. Infected cells were subcutaneously injected into the flanks of 6-week-old male nude mice to induce tumor formation. Tumor diameters were measured at regular intervals, and the tumor volume was measured every 3 days using the following formula: volume = length × width^2^/2. Mice were killed 27 days after injection and tumor grafts were excised, weighed, harvested, fixed, and embedded for histological examination.

### Immunofluorescence staining

For immunofluorescence staining, cells were fixed in 4% paraformaldehyde and incubated in hydrogen peroxide, to inhibit endogenous peroxidase activity. Non-specific antibody-binding sites were blocked with 4% bovine serum albumin for 1 h and cells were labeled overnight with E-cadherin (ab40772, 1:100 dilution) and N-cadherin (ab98952, 1:100 dilution) at 4 °C overnight followed by goat anti-rabbit IgG H&L (Cy3 ®) preadsorbed or goat anti-mouse IgG H&L (FITC)for 2 h at 37 °C. Cells were counterstained with DAPI and observed under a confocal microscope.

### Half maximal inhibitory concentration (IC50) assay

To measure the proportion of viable cells, MTT (3-[4,5-dimethylthiazol-2-Yl]−2,5-diphenyltetrazoliumbromide) staining was used. The dose required to inhibit the metabolic activity of 50% of the cell population (IC50 value) was calculated from logarithmic sigmoidal dose-response curves generated using GraphPad Prism v6 software (GraphPad Inc). For paclitaxel, the IC50 was 101 ng/mL for A2780 cells and 147 ng/mL for SKOV-3 cells.

### Colony formation assay

Cells were seeded at 300 cells/well in 6-well plates and allowed to attach for 24 h. They were then grown for 14 days with or without 101 ng/mL paclitaxel for A2780 cells or 147 ng/mL paclitaxel for SKOV-3 cells. Colonies were fixed and stained with 0.1% crystal violet in 100% ethanol and then counted and captured. Clones that consisted of at least 50 cells were counted as one colony. Cell formation assays were performed in triplicate.

### Paclitaxel treatment

Paclitaxel (Taxol) was purchased from Sigma-Aldrich. The drugs were kept as 1 mg/mL stock solutions in sterile PBS at −20 °C. For paclitaxel content measurement, tumor cells were treated for 7 days at a concentration of 0, 3.125, 6.25, 12.5, 25, 50, 100, 200, 400, and 800 ng/mL. The culture medium was replaced on a daily basis. For the tumor formation in vivo, the nude mice were injected once every 3 days with 5 mg/kg of paclitaxel (dissolved in normal saline)/kg body weight. Controls were treated with the same volume of normal saline.

### Lentivirus construction

To knockdown ST3GAL1, three short hairpin RNA (shRNA) constructs: ST3GAL1-shRNA1# (5′- TAAGAAGACTCCCTCAGGTTG-3′) and ST3GAL1-shRNA2# (5′-AACATCAGCTTCAAACCCTGC-3′), and ST3GAL1-shRNA3# (5′- TTGAGAAGATGACCGAGAGGA-3′) and a scrambled NC shRNA (scr, 5′- GACCTGTACGCCAACACAGTG -3′) were chemically synthesized at Genechem (Shanghai, China), the shRNA sequences were synthesized and cloned into recombinant shRNA expression vectors. The shRNAs were cloned into the PLKO.1-puro recombinant shRNA expression vector (Invitrogen) and co-transfected with the lentiviral packaging helper plasmids, pCMV-dR8.91 and envelope VSV-G (Addgen, Cambridge, MA, USA) into 293 T cells using Lipofectamine 2000 (Invitrogen). Supernatants were collected 72 h after transfection and concentrated by ultracentrifugation. Infected cells were selected by 4 μg/mL puromycin (Invitrogen) for 2 weeks to generate stable-transfected cell lines.

To overexpress ST3GAL1 (ST3GAL1-OE), full length human ST3GAL1 cDNA was amplified and cloned into the pCDH-CMV-MCS-Puro expression vector and the pCDHpPACKH1TM Lentivector Packaging Kit (System Biosciences, Mountain View, CA, USA) was used to produce lentivirus particles. Infected cells were selected by 4 μg/ml puromycin for 2 weeks to generate stable cell lines.

### Statistical analysis

All results are presented as the mean ± standard deviation (SD) from three independent experiments performed in triplicate. Statistical analyses were performed using SPSS statistical software (Version 17.0; SPSS, Inc., Chicago, IL, USA). The Pearson *χ*^2^ test was used to analyze the relationship between ST3GAL1 expression and pathological features. The Student’s *t*-test or one-way ANOVA was used to analyze data. *p* < 0.05 was considered statistically significant.

## Results

### ST3GAL1 is upregulated in ovarian cancer tissues

**Fig. 1:**
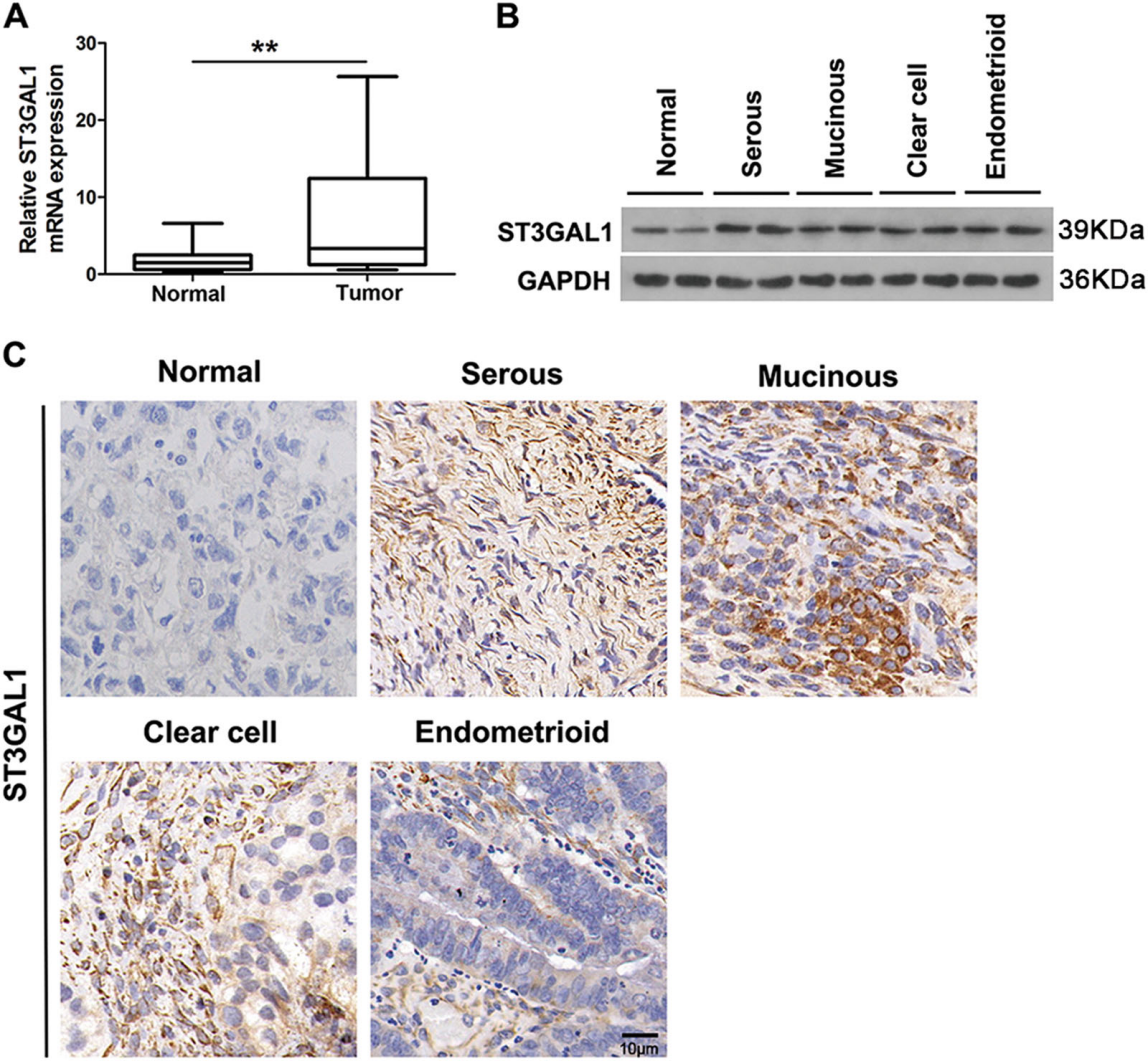
The expression of ST3GAL1 in ovarian cancer tissues and normal tissues. **a** ST3GAL1 expression in 78 ovarian cancer tumor tissues and 15 relative normal tissues analyzed by qRT-PCR (**p < 0.01, Student’s *t*-test). **b** ST3GAL1 protein expression was analyzed by western blots in ovarian cancer tissues. **c** ST3GAL1 expression analyzed by immunohistochemistry in ovarian cancer tissues and normal tissues, respectively

The expression of ST3GAL1 and clinicopathological characteristics were assessed in 78 patients with ovarian cancer (Table 1). Levels of ST3GAL1 were found to be higher in patients with TNM stage III than in those with lower grade ovarian cancer (*p* = 0.014). There was no significant difference in levels of ST3GAL1 in relation to histopathological type (serous, mucinous, clear cell, or endometrioid) or the age of patients, although ST3GAL1 was found at higher levels in serous tissue. The mRNA expression of ST3GAL1 was significantly higher in ovarian cancer tissue than in normal tissue (*p* < 0.01, Student’s *t*-test) (Fig. 1a) and protein levels were also elevated in each histopathological type of cancer compared with normal tissue (Fig. 1b). This finding was confirmed by an increased IHC staining of ST3GAL1 in serous, mucinous, clear cell, or endometrioid tissue when compared to normal ovarian tissue (Fig. 1c).

### ST3GAL1 expression is associated with cell growth, migration, and invasion in vitro

**Fig. 2:**
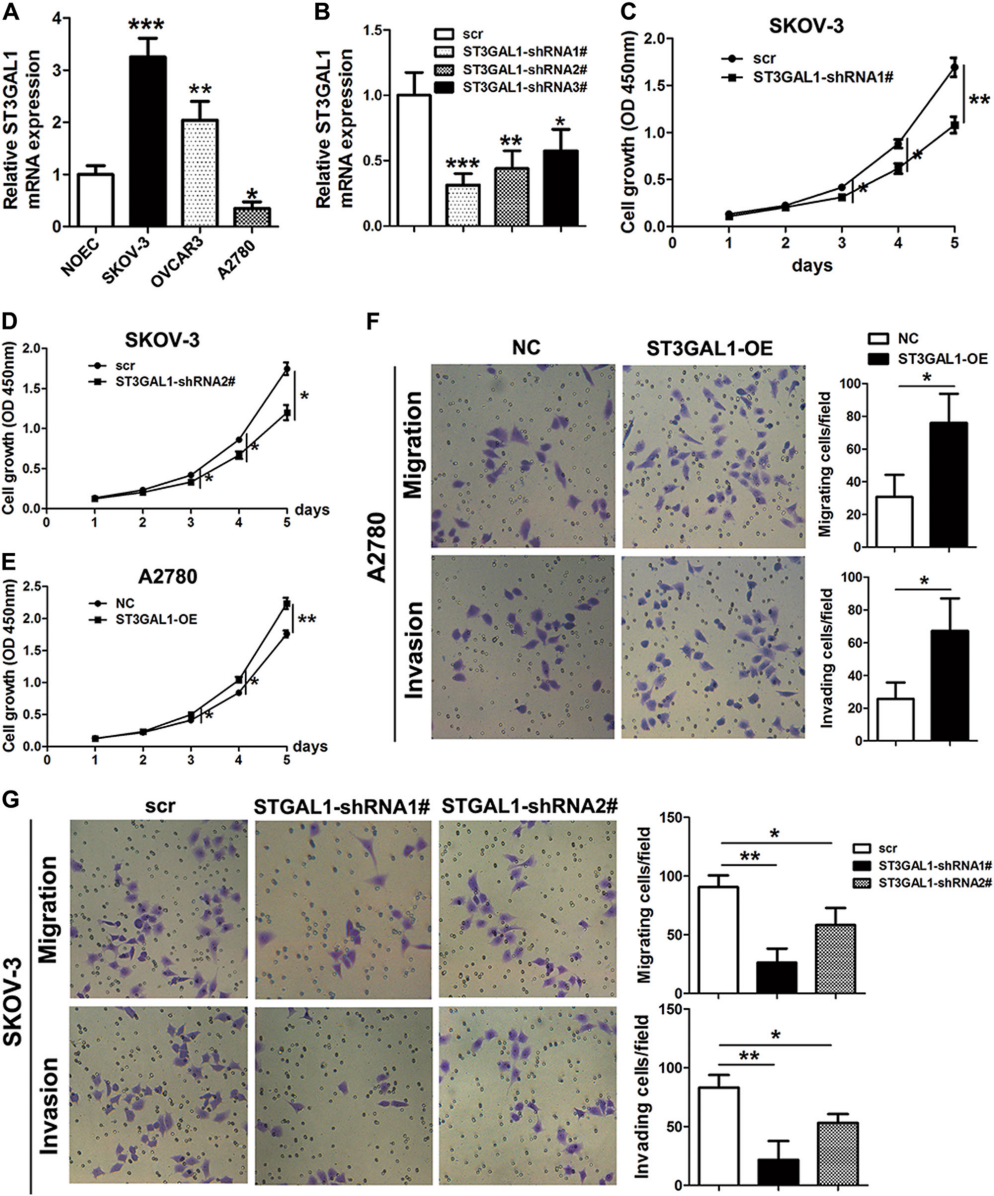
ST3GAL1 promotes cell growth, migration, and invasion in ovarian cancer cells. **a** qRT-PCR analysis of ST3GAL1 expression in the ovarian cancer cell lines SKOV-3, OVCAR3, and A2780 and the normal ovarian cell line NOEC (*p < 0.05, **p < 0.01, and ***p < 0.001, one-way ANOVA). **b** Knockdown of ST3GAL1 (ST3GAL1 shRNA1#–3#) in SKOV-3 cells and qRT-PCR assessment of ST3GAL1 expression (*p < 0.05, **p < 0.01, ***p < 0.001, one-way ANOVA). SKOV-3 cells were transfected with ST3GAL1-shRNA1# or shRNA2# and A2780 cells were transfected overexpression ST3GAL1 (ST3GAL1-OE). **c**–**e** Cell growth was determined by the CCK-8 assay (*p < 0.05, **p < 0.01, one-way ANOVA). **f**, **g** Transwell assay was used to detect cell migration and invasion (*p < 0.05, and **p < 0.01, one-way ANOVA)

After establishing that ST3GAL1 was upregulated in ovarian cancer tissue we assessed its expression in three ovarian cancer cell lines, SKOV-3, OVCAR3, and A2780, and in control NOECs (Fig. 2a). There was a significant difference in the level of ST3GAL1 expression between the three cancer cell lines with the highest expression in SKOV-3 cells and the second highest in OVCAR3 cells. The expression of ST3GAL1 was significantly higher in SKOV-3 and OVCAR3 cells compared with NOECs. However, in A2780 ovarian cancer cells, ST3GAL1 was expressed at a lower level than in normal ovarian cells. To assess whether the expression of ST3GAL1 had the potential to influence proliferation, migration, and invasion in the ovarian cancer cells, we carried out assays with ST3GAL1 over or under-expressed. Three shRNA constructs were created to knockdown ST3GAL1 expression and qRT-PCR analysis was used to measure the relative ST3GAL1 expression in SKOV-3 cells, which is the ovarian cancer cell line with the highest expression of ST3GAL1. The shRNA constructs reduced the expression of ST3GAL1 but at different levels (Fig. 2b). ST3GAL1-shRNA#1 and #2 were used for further studies. When ST3GAL1 expression was reduced by knockdown in SKOV-3 cells, the level of cell proliferation was also reduced (Fig. 2c, d). However, overexpressing ST3GAL1 in A2780 cells increased proliferation (Fig. 2e) and also increased the level of cell migration and invasion (Fig. 2f). Cell migration and invasion were significantly reduced in SKOV-3 cells with ST3GAL1 knockdown and seemed to depend on the level of ST3GAL1 silencing by each shRNA construct (Fig. 2g). Overall our results indicate that overexpressing ST3GAL1 promotes cell growth, migration, and invasion in ovarian cancer cells whereas inhibiting STG3GAL1 expression has the opposite effect on cell growth, migration, and invasion.

### Overexpression of ST3GAL1 enhances while knockdown inhibits TGF-β1-induced epithelial–mesenchymal transition, migration, and invasion in ovarian cancer cells

**Fig. 3:**
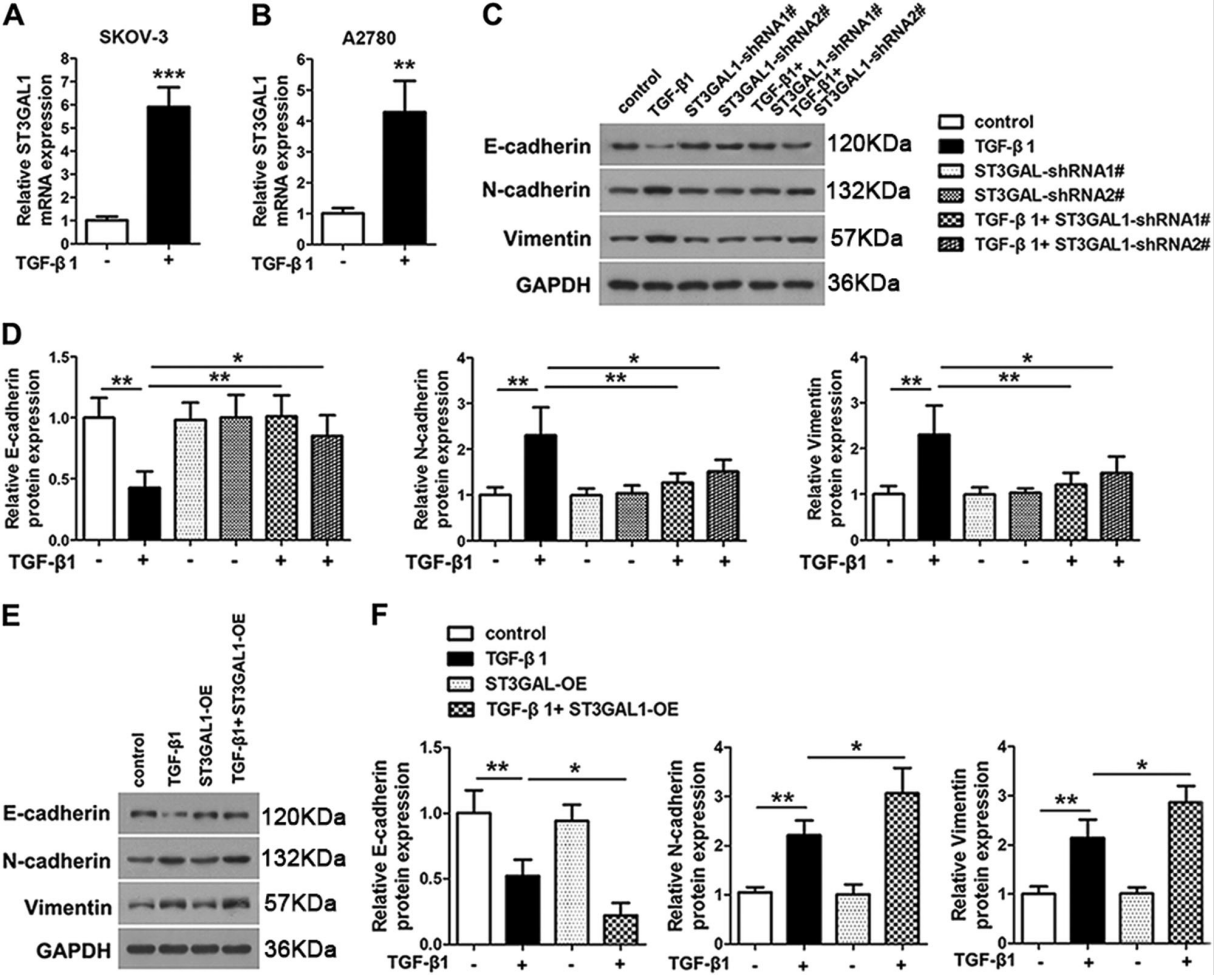
ST3GAL1 regulates epithelial–mesenchymal transition (EMT) protein expression induced by TGF-β1. **a**, **b** SKOV-3 and A2780 cells were treated with or without 10 ng/mL TGF-β1, qRT-PCR analyzed ST3GAL1 mRNA expression (**p < 0.01, Student’s *t*-test). SKOV-3 cells treated with TGF-β1 or transfected with ST3GAL1-shRNA1# or shRNA2#, or both TGF-β1 and ST3GAL1-shRNA1# or shRNA2#, untransfected cells were used as a control. **c, d** E-cadherin, N-cadherin, and Vimentin protein expression were analyzed by western blots. A2780 cells treated with TGF-β1 or transfected with ST3GAL1-OE, or both TGF-β1 and ST3GAL1-OE, untransfected cells were used as a control. **e**, **f** E-cadherin, N-cadherin, and Vimentin protein expression were analyzed by western blots. (*p < 0.05, **p < 0.01, one-way ANOVA)

**Fig. 4:**
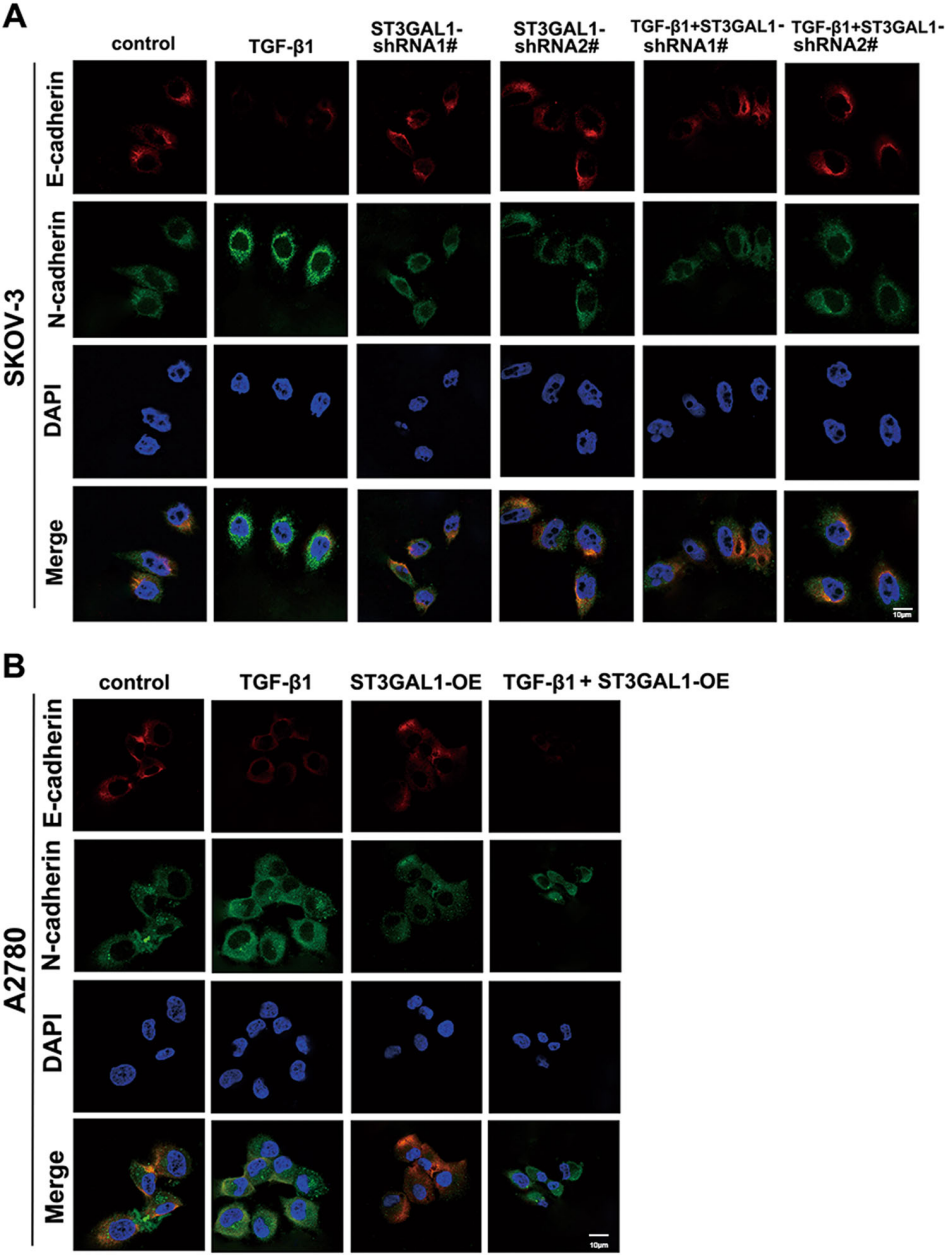
ST3GAL1 regulates E-cadherin and N-cadherin expression induced by TGF-β1. SKOV-3 cells treated with TGF-β1 or transfected with ST3GAL1-shRNA1# or shRNA2#, or both TGF-β1 and ST3GAL1-shRNA1# or shRNA2#, untransfected cells were used as a control. A2780 cells treated with TGF-β1 or transfected with ST3GAL1-OE, or both TGF-β1 and ST3GAL1-OE, untransfected cells were used as a control. **a**, **b** E-cadherin and N-cadherin protein expression were analyzed by cell immunofluorescence

**Fig. 5:**
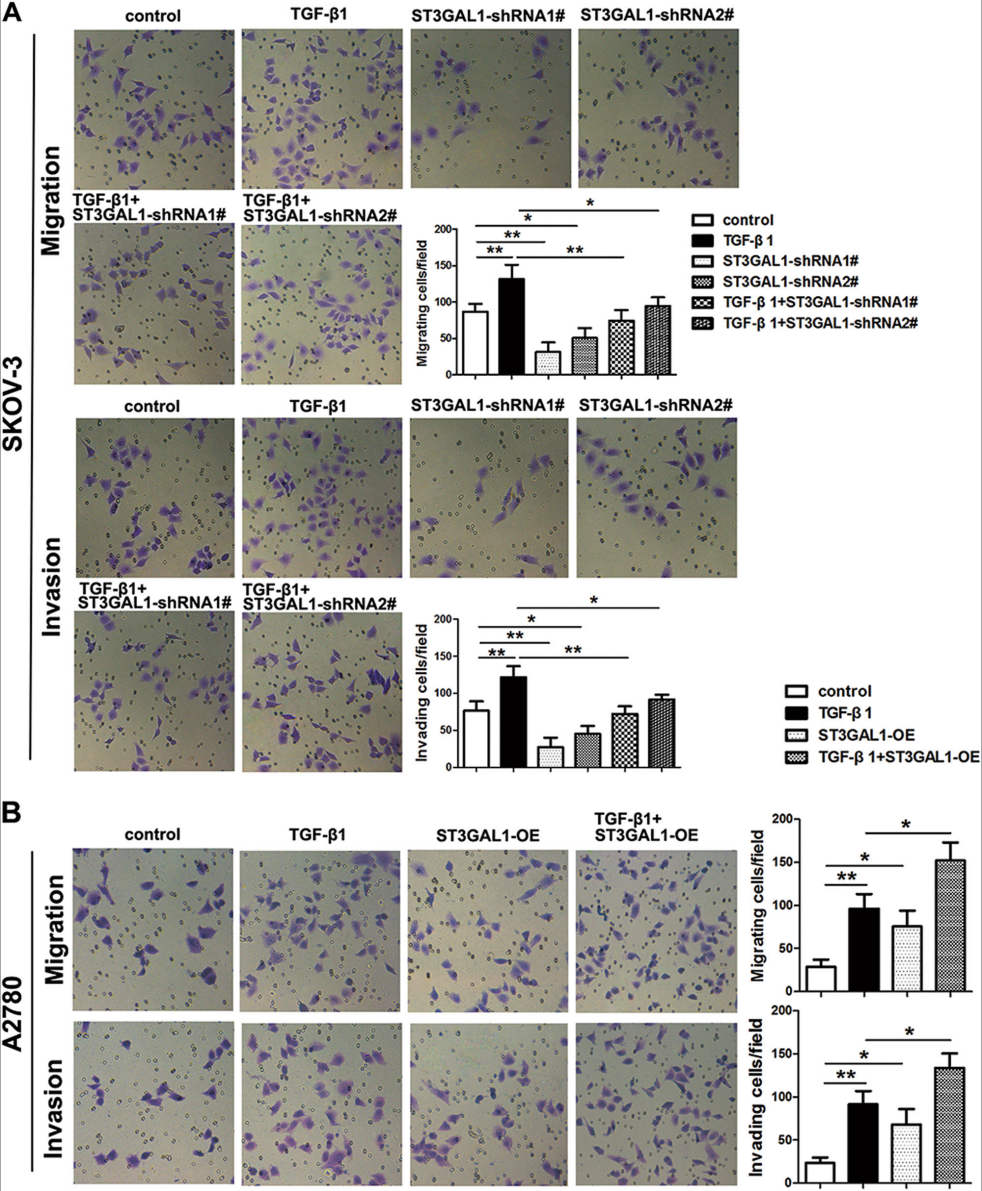
ST3GAL1 impacts cell migration and invasion induced by TGF-β1. SKOV-3 cells treated with TGF-β1 or transfected with ST3GAL1-shRNA1# or shRNA2#, or both TGF-β1 and ST3GAL1-shRNA1# or shRNA2#, untransfected cells were used as a control. A2780 cells treated with TGF-β1 or transfected with ST3GAL1-OE, or both TGF-β1 and ST3GAL1-OE, untransfected cells were used as a control. **a**, **b** SKOV-3 and A2780 cells migration and invasion were analyzed by Transwell assay. *p < 0.05, and **p < 0.01

For a clearer understanding of ST3GAL1 involvement in ovarian cancer, we investigated whether STG3GAL1 participated in EMT. We induced EMT by treating SKOV-3 and A2780 cells with TGF-β1 and found that the mRNA expression of ST3GAL1 was significantly higher in treated cells (Fig. 3a, b). In addition, the protein levels of the EMT markers E-cadherin, N-cadherin, and Vimentin were measured in SKOV-3 cells that were transfected with ST3GAL1-shRNA1# or shRNA2# and cultured with or without TGF-β1 (Fig. 3c, d). The levels of E-cadherin were significantly lower in cells when EMT was induced by TGF-β1 and ST3GAL1 was expressed whereas N-cadherin and Vimentin levels were increased. When ST3GAL1 expression was knocked down the levels of E-cadherin increased while those of N-cadherin and Vimentin were reduced and the presence or absence of TGF-β1 made no significant difference. In fact, when the expression of ST3GAL1 was silenced the protein levels of the EMT markers were similar to those of the untransfected control without TGF-β1 EMT induction, which indicated that EMT had been inhibited. E-cadherin, N-cadherin, and Vimentin levels were also assessed in A2780 cells that were overexpressing ST3GAL1 and cultured with or without TGF-β1 (Fig. 3e, f). Overexpressing ST3GAL1 had the opposite effects of under-expression, E-cadherin levels were reduced by the induction of EMT by TGF-β1 whereas N-cadherin and Vimentin protein levels were increased, indicating that ST3GAL1 plays an integral role in EMT. Cell immunofluorescence of labeled E-cadherin and N-cadherin confirmed the results of ST3GAL1 under-expression or overexpression in either SKOV-3 or A2780 cells, respectively (Fig. 4a, b). Overall these results indicate that ST3GAL1 regulates E-cadherin and N-cadherin expression induced by TGF-β1. The migration and invasion of SKOV-3 cells under-expressing ST3GAL1 and A2780 cells overexpressing ST3GAL1 were also assessed by Transwell assay after treatment with TGF-β1 (Fig. 5a, b). The presence of TGF-β1 increased the number of invading and migrating cells in both SKOV-3 and A2780 cultures. Under-expression of ST3GAL1 in the presence of TGF-β1 reduces invasion and migration whereas overexpression increases invasion and migration. These results indicate that ST3GAL1 can influence the level of cell migration and invasion induced by TGF-β1.

### ST3GAL1 overexpression increases resistance to paclitaxel in vitro and in vivo

**Fig. 6:**
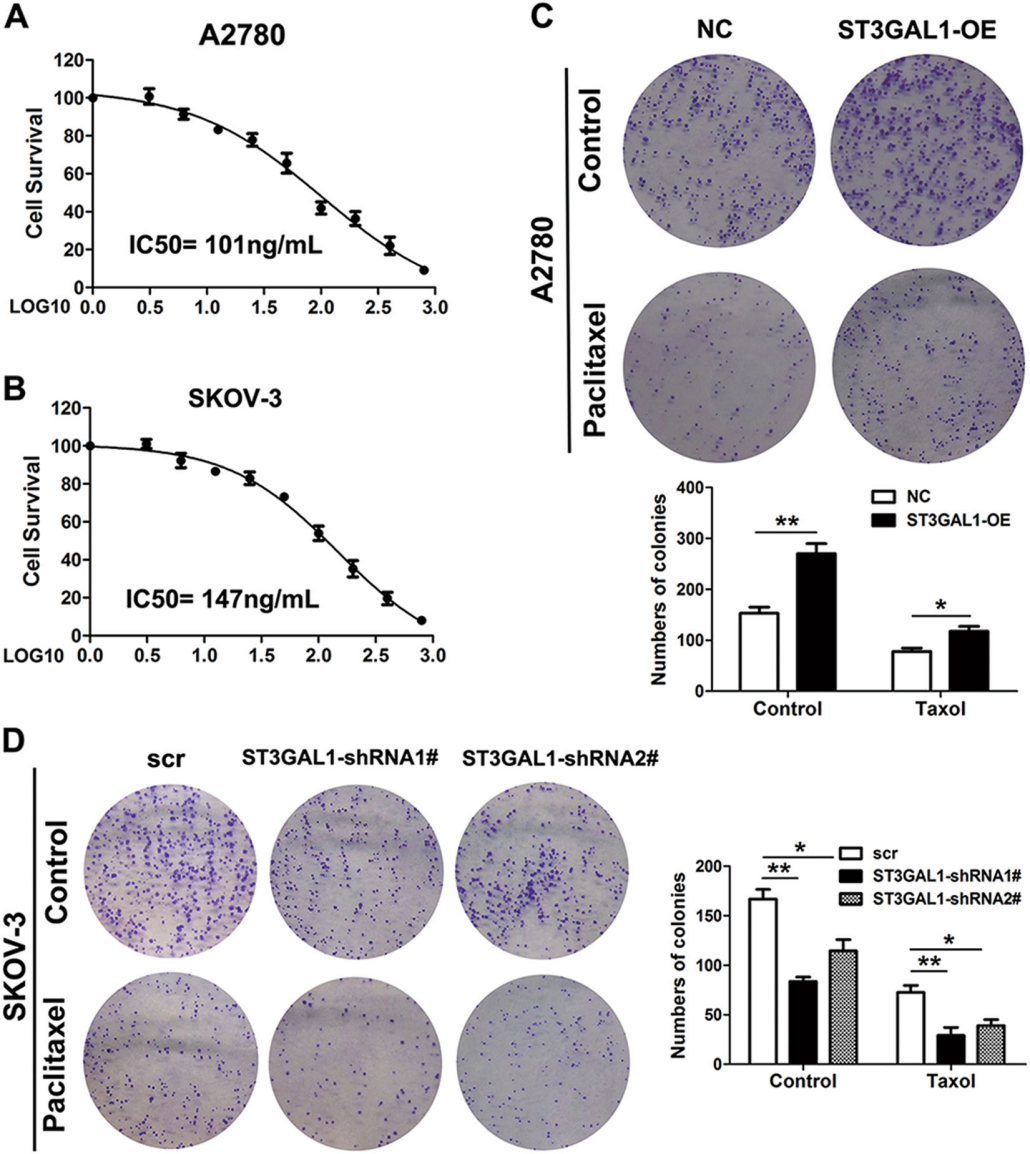
ST3GAL1 increases resistance to paclitaxel (Taxol). **a**, **b** SKOV-3 and A2780 cells were treated with 0, 3.125, 6.25, 12.5, 25, 50, 100, 200, 400, and 800 ng/mL Taxol for 7 days, and the half maximal inhibitory concentration (IC 50) was detected. The IC50 of A2780 is 101 ng/mL and the IC50 of SKOV-3 cells is 147 ng/mL. **c, d** A colony formation assay of A2780 cells transfected with ST3GAL1 (ST3GAL1-OE) for overexpression. SKOV-3 cells transduced with shRNA1# or 2# for ST3GAL1 knockdown. Cells were grown for 14 days under treatment with or without 101 ng/mL Taxol for A2780 cells or 147 ng/mL Taxol for SKOV-3 cells and stained with crystal violet. The colonies were counted and captured. The data represent the mean ± SD from three independent experiments. *p < 0.05, and **p < 0.01

**Fig. 7:**
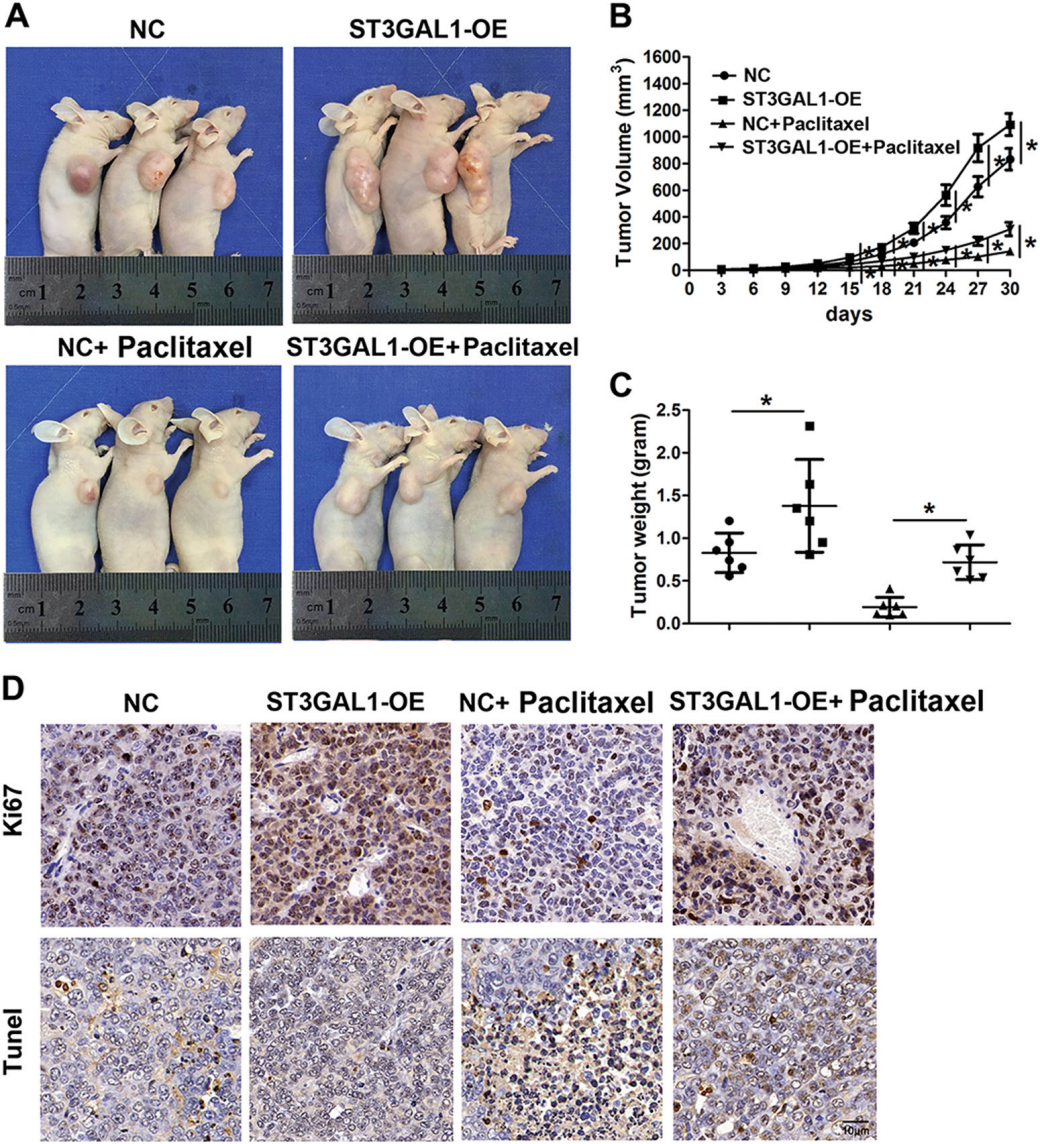
Overexpression of ST3GAL1 reduces the curative effect of paclitaxel (Taxol) on tumor growth in nude mice. **a** The tumor growth curve of overexpression ST3GAL1 A2780 cells was compared with vector-expressing cells after Taxol treatment. Tumor growth was assessed in nude mice that were subcutaneously injected in the right flank with 2.0 × 10^6^ stable transfectants. **b**, **c** Average tumor weight was measured and growth curves were generated (*N* = 6, *p < 0.05, **p < 0.01, ***p < 0.001, one-way ANOVA). **d** Ki67 expression in the four groups was detected by immunohistochemistry, and TUNEL analysis in the four groups

To assess the level of chemoresistance in the ovarian cell lines SKOV-3 and A2780, which had a high and low expression of ST3GAL1, respectively, they were challenged with paclitaxel. The IC_50_ of paclitaxel was deduced for SKOV-3 cells and A2780 cells. The IC_50_ of paclitaxel was higher in SKOV-3 cells (147 ng/mL) than in A2780 cells (101 ng/mL) (Fig. 6a, b). Cells were grown for 14 days with or without the IC_50_ dose of paclitaxel and then stained with crystal violet. The colonies were then counted and captured. Overexpressing ST3GAL1 in A2780 cells increased resistance to paclitaxel as colony formation increased when levels of ST3GAL1 expression increased (Fig. 6c). In contrast, colony formation was reduced in SKOV-3 cells when ST3GAL1 was silenced with shRNA1# or 2# (Fig.6d). These results indicate that overexpression of ST3GAL1 contributes to paclitaxel resistance in ovarian cancer cells. After establishing that ST3GAL1 could increase resistance to paclitaxel in vitro we determined whether a similar effect could occur in vivo using a nude mouse xenograft model. A2780 cells with or without ST3GAL1 overexpressed were injected subcutaneously into mice. The overexpression of ST3GAL1 increased the volume of tumors in mice (Fig.7a–c). Treatment with paclitaxel reduced the tumor volume in mice but to a lesser extent in tumors overexpressing ST3GAL1 (Fig. 7a–c). Ki67 is a cellular marker of proliferation. Ki67 levels were lowest in cells that were treated with paclitaxel but highest in cells overexpressing ST3GAL1 (Fig. 7d). TUNEL analysis found a higher number of apoptotic cells in tumors treated with paclitaxel (Fig. 7d). To conclude, overexpression of ST3GAL1 reduces the curative effect of paclitaxel on tumor growth in nude mice.

## Discussion

Human sialyltransferases are known to be upregulated in several cancers and are also associated with chemoresistance^32–34^. In the present study, we found that ST3GAL1 was upregulated in ovarian cancer tissues and ovarian cancer cell lines. The overexpression of ST3GAL1 increased cell growth, migration, and invasion in vitro whereas under-expression reduced cell growth, migration, and invasion. In addition, TGF-β1 was found to increase ST3GAL1 expression and induce ovarian cell EMT. Moreover, the overexpression of ST3GAL1 increased resistance to paclitaxel in ovarian cancer cells and in a xenograft mouse model.

Other studies have reported changes in the mRNA expression of sialyltransferases in gynecological cancer^35,36^. ST6GAL1 expression was increased in squamous cell carcinoma of the cervix whereas, and in contrast with our results, the expression of ST3GAL1, along with ST3GAL3, and ST3GAL4, was significantly downregulated^35^. Moreover, it was found that high expression of ST6GAL1 was associated with more invasive properties of cervical cancer and that the reduced expression of ST3GAL1, ST3GAL3, and ST3GAL4 may also contribute to the characteristics of cervical cancer^35^. However, in a different study, ST3GAL1 and ST6GAL1 were found to be upregulated in ovarian cancer tissue while the expression of ST3GAL3, ST3GAL4, and ST3GAL6 was downregulated^36^. These results seem to correspond with our results. In addition, we found that the expression of ST3GAL1 could vary significantly in different ovarian histological cell types and there was a significantly higher expression of ST3GAL1 in more severe grades of cancer. ST3GAL1 was significantly upregulated in SKOV-3, which is a human ovarian cancer cell line with resistance to tumor necrosis factor and several cytotoxic drugs^27^. ST3GAL1 was also upregulated in OVCAR3 but not as significantly. The OVCAR3 cell line is derived from progressive ovarian adenocarcinoma and is also resistant to cytotoxic drugs^28^. In contrast, ST3GAL1 was downregulated in A2780 cells. This cell line originates from untreated ovarian cancer cells^27^. Therefore, chemoresistance seems to reflect the level of ST3GAL1 expression in the different cell lines as it is doubtful that an untreated cell line could acquire resistance to cytotoxic drugs. A study on the role of α2,3-sialyltransferases on chemoresistance in chronic myeloid leukemia cell lines, also found that a multidrug-resistant phenotype was associated with the altered expression level of ST3GAL1^34^. Moreover, when the expression of ST3GAL1 was downregulated, sensitivity to adriamycin, paclitaxel, and vincristine increased.

In addition, cancer histological type may influence the expression of ST3GAL1. In tissue samples, we found that a higher number of patients had a high expression of ST3GAL1 in serous ovarian cancer compared with mucinous, clear cell, and endometrioid ovarian cancer. It has been suggested that the increased expression of ST3GAL1 in ovarian serous carcinoma may contribute directly to increased alpha2,3-linked sialylation^36^. Recent research supports our finding that ST3GAL1 expression is increased in serous ovarian cancer and demonstrates that ST3GAL1 may regulate ovarian cancer cell migration and peritoneal dissemination via epidermal growth factor receptor (EGFR) signaling^37^. The EGFR signaling pathway is known to be overexpressed and associated with poor prognosis in more than 70% of ovarian cancer patients^38^. However, in lung cancer, sialylation was found to partially suppress the phosphorylation of EGFR and enhance the sensitivity of a lung cancer cell line to tyrosine kinase inhibitors^39^. Which indicates that the exact characteristics of the sialylation may exert an influence. In ovarian cancer, it was found that in α2,3-linked sialylation was positively correlated with the grade of cancer but α2,6-linked sialylation had no impact on staging or prognosis^40^. Moreover, in clear cell type epithelial ovarian cancer, the upregulated expression of ST3GAL1 and ST3AL4 genes were both associated with reduced E-cadherin expression and increased EMT^40^. ST3GAL1 is mainly associated with O-linked sialylation in cancer cells, whereas ST3GAL4 is mainly associated with N-linked sialylation. When E-cadherin levels are reduced, the adherent junctions between cells become loose and disorganized, which results in metastasis^41^. This is in agreement with our results, the overexpression of ST3GAL1 reduced the level of E-cadherin expression and increased EMT.

Paclitaxel is known to induce apoptosis in various cancers but also initiates a cellular response that leads to chemoresistance. In previous research, paclitaxel-induced caspase-8-dependent apoptosis in SKOV-3 cells but ST3GAL3 was found to increase cellular resistance to paclitaxel^42^. It has been suggested that there could be an interplay between paclitaxel resistance and EMT because both are associated with a common pathway involving glycosylation^43,44^. In the present study, we found that resistance to paclitaxel was associated with reduced levels of E-cadherin and increased cell migration and invasion in relation to the overexpression of ST3GAL1. We also found that ST3GAL1 could influence cell growth and tumorigenicity. ST3GAL1 has also been found to increase cell proliferation and promote tumorigenesis in breast cancer^45^. However, in another study, suppression of α2,3-sialylation in ovarian cancer did not influence tumor cell growth and proliferation but did effect cell motility^40^.

To conclude, in the present study, we found that ST3GAL1 is upregulated in ovarian cancer tissues but the level of ST3GAL1 can vary depending on TNM grade. ST3GAL1 is also upregulated in ovarian cancer cell lines and this seems to be associated with drug resistance. Overexpression of ST3GAL1 increases cell growth, migration, and invasion whereas ST3GAL1 knockdown decreases cell growth, migration, and invasion. Furthermore, TGF-β1 can increase ST3GAL1 expression and induce ovarian cell EMT. However, knockdown of ST3GAL1 inhibits EMT. Finally, overexpression of ST3GAL1 increases resistance to paclitaxel in vitro, and increased cell proliferation and the volume of tumors in a xenograft mouse model. Taken together, our findings have identified a mechanism whereby ST3GAL1 in ovarian cancer could contribute to cytotoxic resistance and promote cell migration. Therefore, ST3GAL1 may be a promising target for overcoming paclitaxel resistance in ovarian carcinoma.
